# Nucleoside triphosphate diphosphohydrolase1 (TcNTPDase-1) gene expression is increased due to heat shock and in infective forms of *Trypanosoma cruzi*

**DOI:** 10.1186/s13071-014-0463-0

**Published:** 2014-10-05

**Authors:** Natália Lins Silva-Gomes, Vitor Ennes-Vidal, Julliane Castro Ferreira Carolo, Marcos Meuser Batista, Maria Nazaré Soeiro, Rubem Menna-Barreto, Otacilio Cruz Moreira

**Affiliations:** Laboratorio de Biologia Molecular e Doenças Endêmicas, Instituto Oswaldo Cruz/ FIOCRUZ, Av. Brasil, 4365. Pavilhão Leônidas Deane, sala 209. Manguinhos, Rio de Janeiro, Brazil; Laboratório de Biologia Celular, Instituto Oswaldo Cruz/ FIOCRUZ, Rio de Janeiro, Brazil

**Keywords:** *T. cruzi*, TcNTPDase-1, Gene expression, Infectivity, Virulence

## Abstract

**Background:**

Ecto-Nucleoside Triphosphate Diphosphohydrolases (Ecto-NTPDases) are enzymes that hydrolyze tri- and/or di-phosphate nucleotides. Evidences point to their participation in *Trypanosoma cruzi* virulence and infectivity. In this work, we evaluate TcNTPDase-1 gene expression in comparison with ecto-NTPDase activity, in order to study the role of TcNTPDase-1 in parasite virulence, infectivity and adaptation to heat shock.

**Findings:**

Comparison between distinct *T. cruzi* isolates (Y, 3663 and 4167 strains, and Dm28c, LL014 and CL-14 clones) showed that TcNTPDase-1 expression was 7.2 ± 1.5 times higher in the Dm28c than the CL-14 avirulent clone. A remarkable expression increase was also observed in the trypomastigote and amastigote forms (22.5 ± 5.6 and 16.3 ± 3.8 times higher than epimastigotes, respectively), indicating that TcNTPDase-1 is overexpressed in *T. cruzi* infective forms. Moreover, heat shock and long-term cultivation also induced a significant increment on TcNTPDase-1 expression.

**Conclusions:**

Our results suggest that TcNTPDase-1 plays an important role on *T. cruzi* infectivity and adaptation to stress conditions, such as long-term cultivation and heat shock.

**Electronic supplementary material:**

The online version of this article (doi:10.1186/s13071-014-0463-0) contains supplementary material, which is available to authorized users.

## Findings

### Background

Chagas disease is a neglected illness caused by the protozoan parasite *Trypanosoma cruzi*, which affects 8 million people in endemic areas of Latin America [1,2]. The different disease spectrums and the course of chronic infection may be consequences of complex interactions between genetic variability of *T. cruzi* subpopulations (classified into TcI to TcVI) [3], host immunogenetics and eco-epidemiological characteristics [3-5]. Current chemotherapy is based on the drugs nifurtimox and benznidazole, which present a lack of effectiveness on the chronic phase of the disease [6]. In this scenario, the search for new drugs and targets to chemotherapy is pivotal.

Ecto-nucleoside triphosphate diphosphohydrolases (Ecto-NTPDases, EC 3.6.1.5) are enzymes that hydrolyze tri- and/or di-phosphate nucleotides [7,8]. Since extracellular ATP is an immune-modulatory molecule that stimulates the secretion of IFN-γ and IL-2 [9], it is hypothesized that ecto-ATPase activity in parasites can be important to the evasion mechanism from the host immune defense, although the mechanism is not clearly elucidated [10]. In 2004, a 2,282 base pair mRNA encoding a full-length NTPDase was cloned, sequenced [11] and named *T. cruzi* NTPDase-1 (TcNTPDase-1; Genbank: AY540630.1), which presents a single copy gene in the genome. In this work, we have developed a quantitative Real-Time RT-PCR assay to quantify TcNTPDase-1 mRNA levels, using TcGAPDH and TcCalmoduline as housekeeping genes (Additional file 1: Figure S1), aiming to contribute to the knowledge about the role of this NTPDase to *T. cruzi* infectivity and virulence. In this sense, we evaluated the TcNTPDase-1 expression in distinct developmental forms (epimastigotes and cell culture-derived amastigotes and trypomastigotes) and parasite isolates, such as the Cl-14, which is described as an avirulent *T. cruzi* clone, since it is unable to promote infection and to induce immune response in a murine model [12,13].

During its life cycle, *T. cruzi* undergoes profound adaptations triggered by a wide range of environmental conditions between the vertebrate or invertebrate hosts, such as variations in pH and temperature. The epimastigote and metacyclic trypomastigote forms interact with the triatomine insect vector at 28°C and the amastigote and trypomastigote forms interact with the mammalian host at 37°C. This heat shock may induce a response from the parasite, promoting the modulation of ecto-ATPase activity [14]. Thus, considering the importance of the ecto-NTPDase activity on the parasite’s purine salvage pathway [15] and heat shock adaptation, the expression of TcNTPDase-1 was also analyzed during *T. cruzi* epimastigote cultivation, at different temperatures, to investigate the expression regulation in response to long-term cultivation or induced by heat shock.

## Methods

### Parasite cultivation

The *T. cruzi* Y, 3663, 4167 strains, and Dm28c, LL014 and CL-14 clones were obtained from the *Coleção de Protozoários da Fundação Oswaldo Cruz* (COLPROT-FIOCRUZ). *T. cruzi* laboratory-adapted epimastigotes were cultivated in BHI medium, supplemented with 10% heat-inactivated fetal bovine serum, at 28°C for 5 days, to reach late-log growth phase.

### Production of culture derived trypomastigotes and amastigotes

The isolation of trypomastigote and amastigote forms was carried out using Vero cells, as detailed elsewhere [16]. Briefly, cell cultures were infected with mice-derived bloodstream trypomastigotes, in a 10:1 parasite/host cell ratio. Infected cells were maintained at 37°C in a 5% CO_2_ atmosphere. After 5–6 days, the supernatant was collected, centrifuged at 500 × g for 5 min, and allowed to stand at 37°C for 30 min for the migration of trypomastigotes into the supernatant. The amastigotes remained in the pellet.

### Ecto-ATPase and ecto-ADPase activity measurements

The extracellular hydrolysis of ATP or ADP by intact parasites was carried out through the measurement of inorganic phosphate (Pi) released in the supernatant, as previously described by De Souza *et al*. [17]. Briefly, ecto-ATPase and ecto-ADPase activities were estimated by the incubation of intact cells (0.5 × 10^8^ parasites) for 1 h at 28°C, in a reaction medium containing 116 mM NaCl, 5.4 mM KCl, 5.5 mM d-glucose, 5 mM MgCl_2_, and 50 mM Hepes–Tris buffer, in the presence of 5 mM ATP or ADP (Sigma-Aldrich), in a final volume of 0.5 mL. The reaction was started by the addition of living parasites and terminated by the addition of 1 mL of ice cold HCl 0.2 M. The cell suspensions were pelleted and supernatant aliquots were used for inorganic phosphate (Pi) quantification [17].

### RNA isolation and cDNA synthesis

Total RNA from *T. cruzi* (1x10^8^ cells) was extracted using TRIzol Reagent (Invitrogen, USA) and treated with DNAse I (Sigma-Aldrich, USA), following manufacturer’s instructions. RNA quantity and purity was estimated by spectrophotometry at 260/280/230 nm. RNA integrity was verified through electrophoresis on a 1.5% (w/v) agarose gel. All reverse transcriptase reactions were performed from 3 μg of RNA using a Superscript III First-strand System (Invitrogen, USA), according to the manufacturer’s instructions.

### -NTPDase-1 gene expression quantification by Real-Time RT-PCR

Real-time quantitative PCR assays were performed in ABI Prism 7500 fast sequence detection system using Power SYBR Green PCR mastermix (Applied Biosystems, USA). The following primers and concentrations were used: *TcNTPDase-I Fw* (600 nmol/L), 5’-GCGGAACCGCAACACCCTCA-3’; *TcNTPDase-I Rv* (600 nmol/L), 5’-CGGTCGAGCTGAAGCGCCAA-3’; *TcCalmoduline* Fw (600 nmol/L), 5’-CCCGACGGAGGCGGAGCTGC-3’; *TcCalmoduline* Rv (600 nmol/L), 5’-GTCCACGTCGGCCTCGCGGA-3’; *TcGAPDH* Fw (300 nmol/L), 5’-GTGCGGCTGCTGTCAACAT-3’; and *TcGAPDH* Rv (300 nmol/L), 5’-AAAGACATGCCCGTCAGCTT- 3’. The conditions for the RT-qPCR were as follows: 95°C for 10 minutes, followed by 40 cycles at 95°C for 15 seconds and 62°C for 1 minute. To monitor the primers specificity, melting curves were performed after each experiment, resulting in a single peak. Reactions were performed in duplicates using 2 μL of cDNA template, in a total volume of 20 μL. The relative quantitative measurement of target gene levels was performed using the ΔΔCt method [18]. As endogenous housekeeping control genes, *T. cruzi* Calmoduline and GAPDH genes were used. PCR assays were in triplicate and data were pooled.

### Statistical analysis

All experiments were performed at least in biological triplicates and experimental duplicates. Data are expressed as arithmetic mean ± Standard Deviation. Student’s *t* test or Mann-Whitney Rank-Sum test were adopted to analyze the statistical significance of the apparent differences. All statistical tests were performed with SigmaPlot for Windows Version 12 (Systat Software). Differences were considered statistically significant when *p <* 0.05.

### Ethical approval

In order to perform the experimental infections with *Trypanosoma cruzi*, swiss mice obtained from the animal facilities of the Oswaldo Cruz Foundation (CECAL/Fiocruz, Rio de Janeiro, Brazil) were housed under specific pathogen free conditions in a 12-hour light-dark cycle with access to food and water *ad libitum*. Our protocols were approved by the Institutional Committee for Animal Ethics of Fiocruz (CEUA/Fiocruz, License LW-16/14).

## Results and discussion

There are lines of evidence that NTPDases are related to virulence and infectivity in protozoan parasites [19-23]. However, most of the studies reported ecto-NTPDase enzymatic activities in intact parasites or plasma membrane fractions. Taking into account that plasma membranes share distinct ecto-nucleotidase activities, such as the Mg^2+^-dependent and Mg^2+^-independent ecto-ATPase activities [23], there is a lack of information regarding the specific contribution of each enzyme to these processes. It is possible that distinct ecto-enzymes could contribute to the parasite adaptation to stress conditions, as nutrients starvation or heat shock. Recently, Mariotini-Moura *et al*. [24] performed heterologous expression, purification and molecular characterization of TcNTPDase-1. By using specific polyclonal antibodies, they confirmed the presence of TcNTPDase-1 not only on the surface of *T. cruzi,* but also in the kinetoplast, nucleus, intracellular vesicles, flagellum and flagellum insertion region. The two latter localizations suggest that the enzyme may have a role in nutrient acquisition. It was also shown that the treatment of the parasite with anti-TcNTPDase-1 antibody decreases adhesion of *T. cruzi* to Vero cells, corroborating the importance of this enzyme to parasite-vertebrate host interaction.

**Figure 1:**
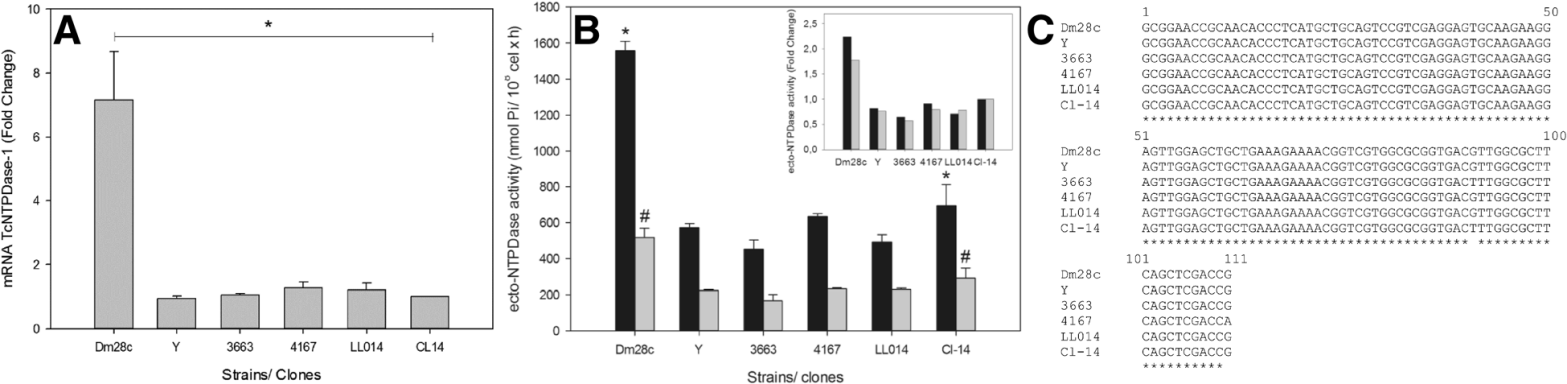
**TcNTPDase-1 expression in epimastigote forms from distinct** ***T. cruzi*** **isolates. A**. TcNTPDase-1 mRNA levels estimated by RT-qPCR. The relative quantification by ∆∆Ct method was performed using the avirulent Cl-14 clone as calibrator. **B**. Ecto-ATPase and ecto-ADPase activities between distinct *T. cruzi* strain/clones. The ecto-nucleotidase activities were estimated in *T. cruzi* epimastigotes, using 5 mM ATP or ADP as substrate. The inset shows the ecto-nucleotidase activities represented as Fold Change versus Cl-14 clone. **C**. Alignment of DNA sequence from PCR products for the TcNTPDase-1. The asterisks indicate identity between the nucleotides. *, # p < 0.05 (versus Cl-14 clone, Mann-Whitney Rank-Sum test).

Therefore, we used a gene expression quantification approach to evaluate the specific contribution of TcNTPDase-1 to parasite virulence, infectivity and adaptation to heat shock. To evaluate gene expression of TcNTPDase-1 between distinct *T. cruzi* isolates, we selected representative strains or clones isolated from mammalian or invertebrate hosts, belonging from TcI to TcVI (Table 1). The TcNTPDase-1 mRNA levels were quantified in comparison to avirulent Cl-14 *T. cruzi* clone (TcVI). The expression of TcNTPDase-1 was 7.2 ± 1.5 times higher for the Dm28c clone (Tc I), than all other isolates, which showed similar low expression levels, like the avirulent Cl-14 clone (Figure 1A). To investigate if the increase in TcNTPDase-1 mRNA levels would result in higher ecto-nucleotidase activities, the ecto-ATPase and ecto-ADPase activities were estimated in intact epimastigotes (Figure 1B). Similar to mRNA levels, the ecto-ATPase and ecto-ADPase activities were also higher for the Dm28c clone than the avirulent Cl-14 clone. However, these increases (2.8 and 1.3 times higher to the ecto-ATPase and ecto-ADPase activities, respectively) were slightly lower than observed with the ecto-NTPDase-1 mRNA. Taking into account that, when using living epimastigotes, extracellular nucleotide hydrolysis might be correlated to different ecto-enzyme activities, and post-translational regulation can also influence the level of active proteins, a significant increase in both mRNA and ecto-NTPDase activity levels suggests that the positive modulation of TcNTPDase-1 expression in epimastigotes from Dm28c clone occurred both at gene and protein levels. To exclude the possibility that the existence of polymorphisms at the TcNTPDase-1 gene could affect the gene expression analysis between these samples, PCR products for the TcNTPDase-1 were sequenced. The DNA alignment indicated a high level of identity (98.2%) between them (Figure 1C). Comparison between epimastigotes forms of the distinct strain/clones suggested that the higher Dm28c TcNTPDase-1 expression could be associated to the parasite interaction with the invertebrate host, but other experiments should be performed for better evaluation. In fact, Dm28c is a *T. cruzi* clone with an elevated rate of colonization of the *Rhodnius prolixus* intestine, although it presents low virulence to the vertebrate host.

**Table 1:** **Identification and classification of** ***T. cruzi*** **strains/clones used in this work**

**Strain/Clone**	DTU*	**Origin**	**Host**
**Dm28c**	*T. cruzi* I	Carabobo, Venezuela	*Didelphis marsupialis*
**Y**	*T. cruzi* II	São Paulo, Brazil	*Homo sapiens*
**3663**	*T. cruzi* III	Amazonas, Brazil	*Panstrongylus geniculatus*
**4167**	*T. cruzi* IV	Amazonas, Brazil	*Rhodnius brethesi*
**LL014**	*T. cruzi* V	Chaco, Argentina	*Triatoma infestans*
**CL-14**	*T. cruzi* VI	Rio Grande do Sul, Brazil	*Triatoma infestans*

*DTU: Discrete Typing Unit.

**Figure 2:**
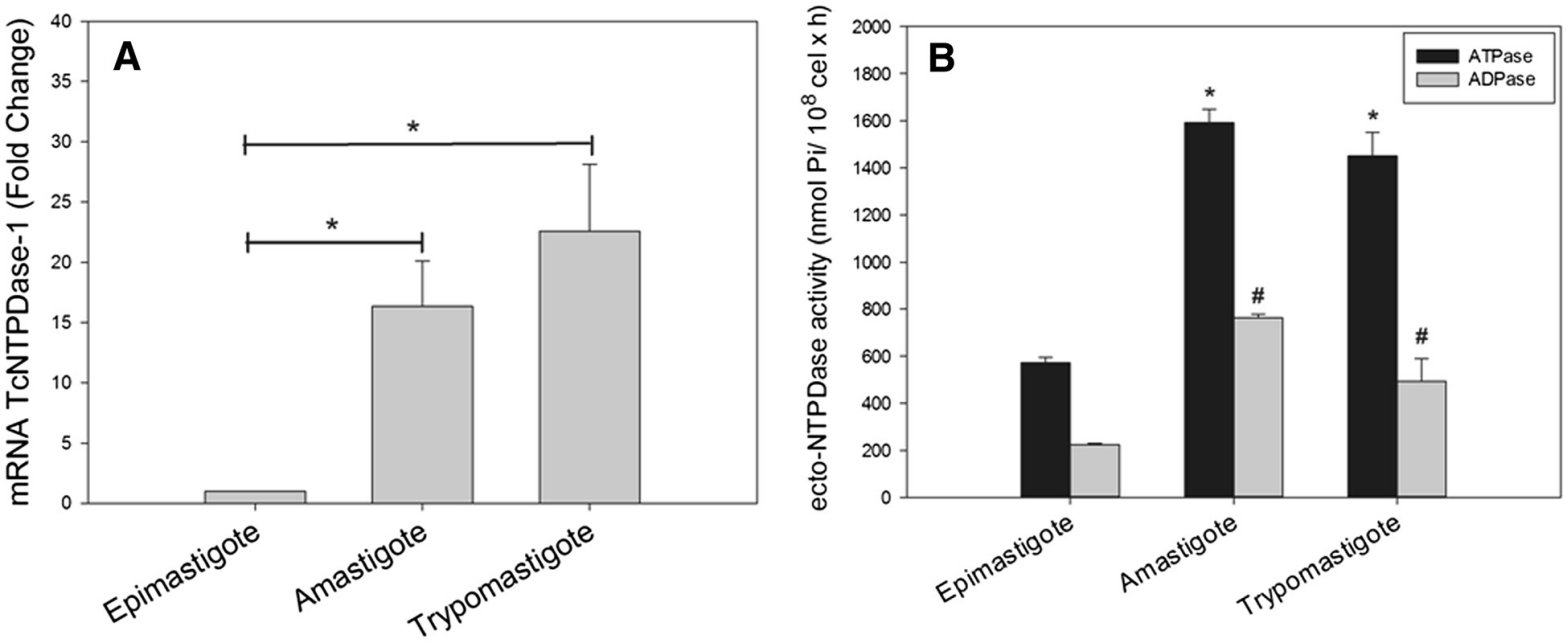
**TcNTPDase-1 expression in infective and non-infective forms of** ***T. cruzi*** **. A**. TcNTPDase-1 mRNA levels in epimastigotes, amastigotes and trypomastigotes, estimated by RT-qPCR. The relative quantification by ∆∆Ct method was performed using the epimastigote form as calibrator. **B**. Ecto-ATPase and ecto-ADPase activities in distinct developmental forms. The ecto-nucleotidase activities were estimated in epimastigotes, amastigotes and trypomastigotes, using 5 mM ATP or ADP as substrate.*, #p < 0.05 (versus epimastigotes, Mann-Whitney Rank-Sum test).

To investigate TcNTPDase-1 expression in infective parasite forms, we obtained cell-derived trypomastigotes and amastigotes, through the infection of Vero cells with bloodstream-derived trypomastigotes (Y strain). TcNTPDase-1 mRNA levels (Figure 2A) and ecto-NTPDase activities (Figure 2B) were estimated and compared between developmental forms. A remarkable increase in the TcNTPDase-1 mRNA levels related to trypomastigote and amastigote forms was observed (22.5 ± 5.6 and 16.3 ± 3.8 times higher than epimastigotes, respectively). The ecto-ATPase and ecto-ADPase activities were also significantly higher in trypomastigotes and amastigotes. The ectoATPase was 2.9 and 2.8 times higher and the ecto-ADPase was 3.0 and 3.4 times higher in tripomastigotes and amastigotes, respectively, when compared to the non-infective epimastigote form. Accordingly, we observed a simultaneous increase in mRNA and ecto-NTPDase activities in infectant forms of *T. cruzi*, suggesting the positive modulation of TcNTPDase-1 expression both at gene and protein levels. It corroborates with previous data obtained by Meyer-Fernandes *et al.* [23], which described increased Mg^2+^-dependent ecto-ATPase activities in trypomastigote and amastigote forms. The positive modulation of TcNTPDase-1 expression corroborates with the hypothesis that this enzyme plays an important role in *T. cruzi* infectivity.

**Figure 3:**
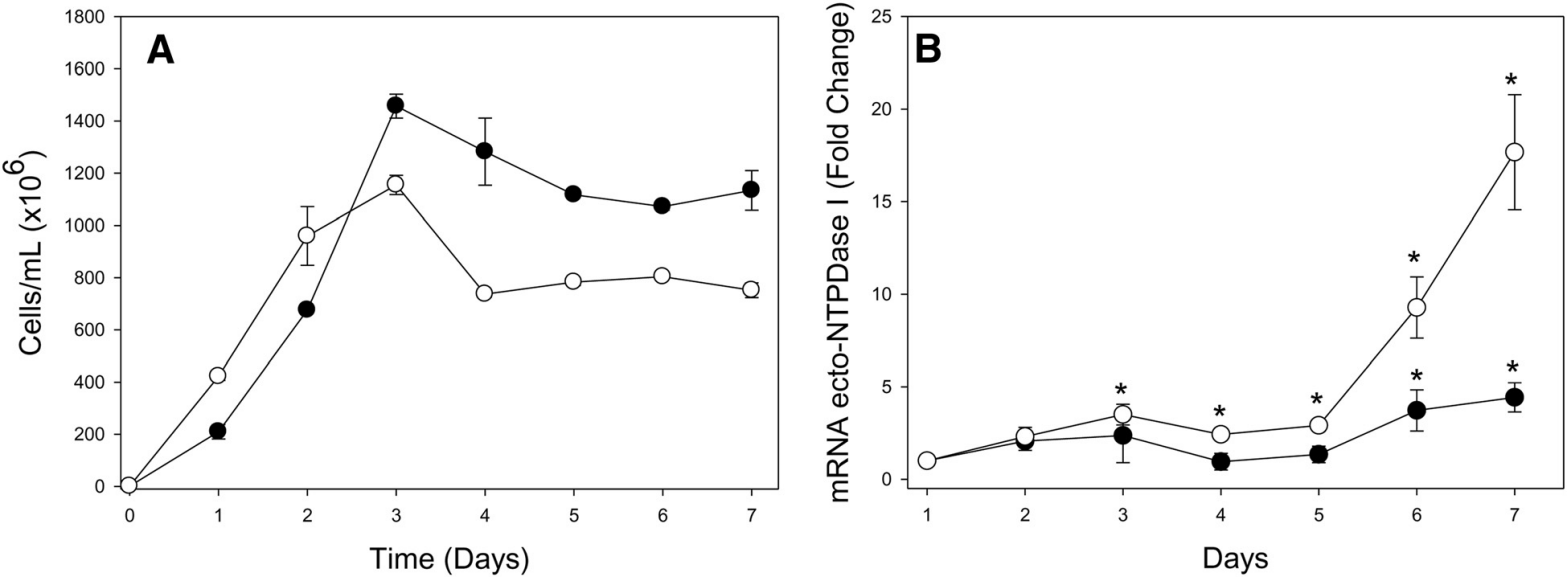
**TcNTPDase-1 gene expression during epimastigote** ***in vitro*** **cultivation and induced by heat-shock. A**. Growth curve of *T. cruzi* epimastigotes, in BHI medium, at different temperatures. (●) 28°C, (●) 37°C. **B**. TcNTPDase-1 mRNA levels during *T. cruzi* growth curve. The relative quantification by ∆∆Ct method was performed using epimastigotes (Y strain) from the first day of cultivation as calibrator. (●) 28°C, (●) 37°C. *p < 0.05 (versus day 1, Student’s *t* test).

During its life cycle, *T. cruzi* is exposed to different environments and temperatures between insect and mammalian hosts (28 and 37°C, respectively). Parasite exposition to 37°C induces an overexpression of various proteins, including heat shock proteins, which may be important to parasite invasion and proliferation [25,26]. Therefore, in order to evaluate the modulation of TcNTPDase-1 gene expression during cellular growth and in response to heat shock, in a strain that expresses low levels of Tc-NTPDase-1 in epimastigote forms, parasites from Y strain were cultivated for 7 days at 28°C and 37°C (Figure 3A). At 28°C, we observed a slight but significant increase at TcNTPDase-1 mRNA level by the sixth and seventh days after the inoculum (3.7 ± 1.1 and 4.4 ± 0.8 times higher than day 1, respectively). In contrast, at 37°C, the expression was 17.7 ± 3.1 times higher, at the seventh day after the inoculum, suggesting that heat shock and long-term cultivation could increase TcNTPDase-1 gene expression (Figure 3B). Similar data were observed for the protozoan parasite *Trichomonas vaginalis* by Frasson *et al*. [27]. These authors described the higher NTPDase expression levels in parasites cultivated in a limited serum supplementation condition, particularly for the clinical isolate. In conjunction, our results suggest the role of TcNTPDase-1 on *T. cruzi* infectivity and adjustment to stress conditions such as nutrients starvation and heat shock. Due to its importance for *T. cruzi*, further studies should be performed to investigate the role of TcNTPDase-1 on the parasite virulence, in order to consider the possibility to evaluate this enzyme as a new candidate target for Chagas Disease chemotherapy.

## Additional file

Additional file 1: Figure S1. Standardization of Real-Time RT-qPCR for the *T. cruzi* ecto-NTPDase I gene expression analysis. A. Representative amplification curves for the TcNTPDase-1, GAPDH and Calmoduline targets. B. Representative melting curves for the TcNTPDase-1, GAPDH and Calmoduline targets. C. Validation of the ∆∆Ct method for GAPDH and Calmoduline as housekeeping genes. Slopes: 0.1 (TcNTPDase-1 GAPDH) and -0.06 (TcNTPDase-1-Calmoduline).
