# Supplementary figures and images for: Nucleoside triphosphate diphosphohydrolase1 (TcNTPDase-1) gene expression is increased due to heat shock and in infective forms of Trypanosoma cruzi
Source: Parasit Vectors. 2014 Oct 5;7:463. doi: 10.1186/s13071-014-0463-0 (PMC4210531; doi:10.1186/s13071-014-0463-0)

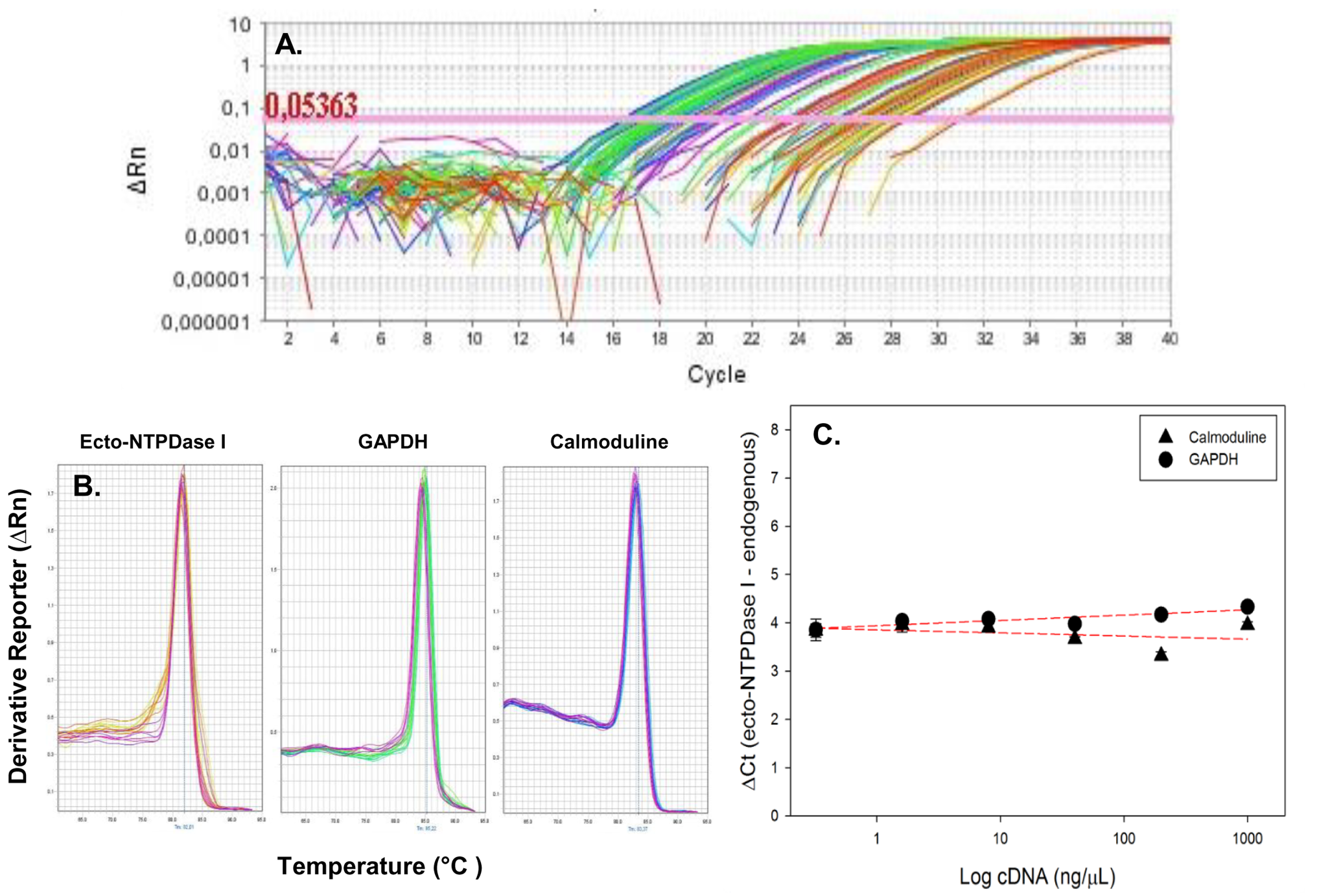

Supplement: Additional file 1: Figure S1. — Standardization of Real-Time RT-qPCR for the T. cruzi ecto-NTPDase I gene expression analysis. A. Representative amplification curves for the TcNTPDase-1, GAPDH and Calmoduline targets. B. Representative melting curves for the TcNTPDase-1, GAPDH and Calmoduline targets. C. Validation of the ∆∆Ct method for GAPDH and Calmoduline as housekeeping genes. Slopes: 0.1 (TcNTPDase-1 GAPDH) and -0.06 (TcNTPDase-1-Calmoduline). [file 13071_2014_463_MOESM1_ESM.tiff]
